# Introduction and evaluation of a clinical compulsory elective course on domestic violence

**DOI:** 10.3205/zma001577

**Published:** 2022-11-15

**Authors:** Paulina Juszczyk, Lisa Sondern, Bettina Pfleiderer

**Affiliations:** 1 University of Muenster, Clinic for Radiology and Medical Faculty, Muenster, Germany

**Keywords:** domestic violence, training platform, student course, medical teaching

## Abstract

**Goal:**

Knowledge about domestic violence (DV) in medicine is often lacking, even though health professionals are often the first point of contact for victims of DV. A clinical compulsory elective course for medical students on DV was introduced to increase competences and knowledge on DV. The course is based on the didactic concept of the IMPRODOVA training platform [https://training.improdova.eu/en/] and was first piloted at the Medical School at the University of Muenster in summer 2020. The course was evaluated to assess whether it is suitable to increase knowledge and competences on DV. Accordingly, the following research questions were assessed: What competences and knowledge do students have about DV in general and how do students’ competences and knowledge about DV change after participating in the course?

**Methods:**

Knowledge assessment on DV in general was based on two surveys conducted at the German universities of Muenster and Luebeck in 2020 and 2021. 54 medical students from Muenster (n=37) and Luebeck (n=17) participated. Muenster medical students were asked to complete a questionnaire within a two-week time period prior participation in the clinical compulsory elective course on DV. Luebeck medical students who had registered for a workshop on DV participated in the same survey prior to the webinar. 28 of the medical students in Muenster underwent in addition a post course assessment. The surveys were created using Questback's online survey research tool EFS using a 5-Point-Likert-Scale. Participation was voluntary and anonymous. The results are reported descriptively and differences between pre- and post-surveys were assessed by t-tests and effect sizes.

**Results:**

Knowledge assessment indicated that medical students had severe gaps in knowledge related to DV. Completion of an elective course has contributed to a significant learning and competence progress of the students in all DV subject areas.

**Conclusion:**

The newly established course is suitable to increase knowledge and competences on DV in medical students and should be included mandatory into the medical curriculum.

## Zusammenfassung

**Ziel:**

Das Wissen über das Thema häusliche Gewalt ist in der Medizin oft unzureichend, obwohl Fachkräfte im Gesundheitswesen oft die erste Anlaufstelle für Opfer von häuslicher Gewalt sind. Es wurde daher ein Wahlpflichtkurs für Medizinstudierende im klinischen Teil des Studiums zum Thema häusliche Gewalt eingeführt, mit dem Ziel, die Kompetenzen und das Wissen über häusliche Gewalt zu verbessern. Der Kurs basiert auf dem didaktischen Konzept der IMPRODOVA-Trainingsplattform [https://training.improdova.eu/de/] und wurde im Sommer 2020 erstmals an der Medizinischen Fakultät der Universität Münster erprobt. Der Kurs wurde zudem evaluiert, um zu prüfen, ob er geeignet ist, Wissen und Kompetenzen über häusliche Gewalt zu erweitern. Es wurden die folgenden Forschungsfragen untersucht: Welche Kompetenzen und welches Wissen haben die Studierenden über häusliche Gewalt im Allgemeinen und wie verändern sich die Kompetenzen und das Wissen der Studierenden über häusliche Gewalt nach der Teilnahme an dem Kurs?

**Methodik:**

Die Evaluation des Wissens über häusliche Gewalt im Allgemeinen basierte auf zwei Umfragen, die an zwei deutschen Universitäten in Münster und Lübeck in den Jahren 2020 und 2021 durchgeführt wurden. 54 Medizinstudierende aus Münster (n=37) und Lübeck (n=17) nahmen daran teil. Die Münsteraner Medizinstudierenden wurden gebeten innerhalb eines zweiwöchigen Zeitraums vor der Teilnahme an dem klinischen Wahlpflichtkurs zu häuslicher Gewalt einen Fragebogen auszufüllen. Lübecker Medizinstudierende, die sich für einen Online-Workshop zum Thema häusliche Gewalt angemeldet hatten, nahmen vor dem Webinar an der gleichen Umfrage teil. Zusätzlich nahmen 28 Medizinstudierende aus Münster an einer Befragung nach dem Kurs teil. Die Umfragen wurden mit dem Online-Umfragetool EFS von Questback unter Verwendung einer 5-Punkte-Likert-Skala erstellt. Die Teilnahme war freiwillig und anonym. Die Ergebnisse wurden deskriptiv ausgewertet und die Unterschiede zwischen Vor- und Nachbefragung mittels t-Tests und Effektgrößen analysiert.

**Ergebnisse:**

Die Auswertung der vorhandenen Kenntnisse ergab, dass die Medizinstudierenden große Wissenslücken in Bezug auf häusliche Gewalt hatten. Die Absolvierung des Kurses trug zu einem erheblichen Lern- und Kompetenzfortschritt der Studierenden in allen Themenbereichen zu häuslicher Gewalt bei.

**Schlussfolgerung:**

Der neu eingeführte Kurs ist geeignet, um das Wissen und die Kompetenzen über häusliche Gewalt bei Medizinstudierenden zu verbessern und sollte verpflichtend in den medizinischen Lehrplan aufgenommen werden.

## 1. Introduction

Health professionals play a major role in the detection and intervention of Domestic Violence (DV) as they are often the first point of contact for victims of DV. DV is defined as violence within the family, against children, spouses and elderly family members [1]. Seriousness of DV is related to intensity, duration, and consequences of violence that may occur causing physical, sexual or psychological harm, including physical aggression, sexual coercion, psychological abuse and controlling behaviour [2]. Women are more likely to experience repeated and severe forms of DV. Globally, one in three women has experienced physical and/or sexual violence since the age of 15 years [1].

Resulting short and long-term health consequences prompt victims to seek help from the medical profession, however DV is systematically underreported, as health professionals seem to have not enough knowledge and competences to detect DV victims:

Interviews with professionals from the medical sector within the IMPRODOVA project indicated that they are not sufficiently trained regarding DV matters and not aware of their role as frontline responder in cases of DV [3]. As knowledge about DV in general, symptoms and red flags are often not part of the mandatory curriculum for medical students in most European countries as IMPRODOVA research indicated [3], many health professionals are not aware of the important role they play. Primarily, they see their role in taking care of the medical needs of their patients that might be consequences of DV. They rarely consider themselves as frontline responders to DV and do not focus sufficiently on the other needs of the victim besides medical care (e.g., offering information or help, assessing risk for further harm). To better identify patients as victims and to support them, a better understanding of their own role, but also of the roles of other frontline responders is necessary. First studies show good results regarding the identification of DV victims after appropriate trainings (e.g., [4]).

As knowledge on DV in medicine is low and the topic of DV is not integrated at German medical faculties [3], the clinical compulsory elective course “Domestic violence in an international context” was newly developed for medical students piloted at the medical faculty of the University of Muenster, Germany as part of the European Union funded project IMPRODOVA to increase knowledge and competences on DV to close this gap [5], [6]. The course integrates not only the German but also the European perspective, because epidemiological data on the prevalence of DV in Europe indicate that DV is a global problem and that prevalence, indicators and underlying reasons across Europe are comparable and cultural aspects also need to be taken into account [7].

The course builds on the didactic concept and materials for the medical sector from the IMPRODOVA training platform which is modular and consists of seven modules for three main frontline responder groups: the police, the health sector and professionals from NGOs and the social sector [5]. The modules are thematically consistent, but with the content adapted to the respective sector. In addition to the specific training modules, data and statistics are presented for all groups in a separate section: it includes information about victimisation surveys and police data in the European Union. In addition, training videos, case studies and scenario-based learning, knowledge assessments, and downloadable factsheets and presentations can be found in the various modules or can be selected separately from the teaching materials section.

## 2. Project description

### 2.1. The IMPRODOVA project and its methodology

The IMPRODOVA project [https://improdova.eu/] – Improving Frontline Responses to High Impact Domestic Violence – is a research and innovation project funded by the European Union (grant No 787054) involving partners from eight EU countries [8].

IMPRODOVA started in May 2018 and ended in August 2021. IMPRODOVA started with the analysis of policy implementation, legislation, data, risk assessment, case documentation, intersectoral cooperation, and trainings, including the status quo of the medical profession related to DV. Next, 296 interviews with different frontline responders across Europe representing the police, the health sector, and the social sector provided information on the extent standards are integrated into the daily work of frontline responders [9], [10] (see deliverable report D2.2 on the cross-national comparison on the implementation of international norms and national best practices of frontline responders [11]. Based on that, the project developed innovative toolkits (e.g., a training platform with various training materials to improve a multi-agency collaboration).

#### 2.2. Concept of the clinical compulsory elective course “domestic violence in an international context”

The overarching aim of the elective course “domestic violence in an international context” is to make students competent to ask patients about DV and to support those of them who are victims of DV. This requires competences that include also aspects such as:

- forms of DV
- gendered risk factors in cases of DV
- sex and gender aspects in risk assessment [10] in cases of DV
- consequences of DV
- indicators of DV
- communication in cases of DV and what happens after a disclosure
- medical assessment and securing of evidence
- risk assessment and safety planning in cases of DV
- international standards and legal frameworks in Europe and Germany
- inter-agency cooperation in cases of DV
- radiological indicators which contribute to the documentation of the extent of physical injuries. In cases of DV, patients have a higher frequency of potential violence-related imaging findings, such as acute fractures [12], in addition to obstetric-gynaecologic findings

Based on this, an exemplary course agenda was developed which can be modified or amended by other universities at any time. The agenda of the elective course is part of the IMPRODOVA training materials and can be found on the corresponding training platform [https://training.improdova.eu/en/training-materials-for-the-health-sector/workshop-and-student-courses/] and in the attachment 1 . 

**Figure 1:**
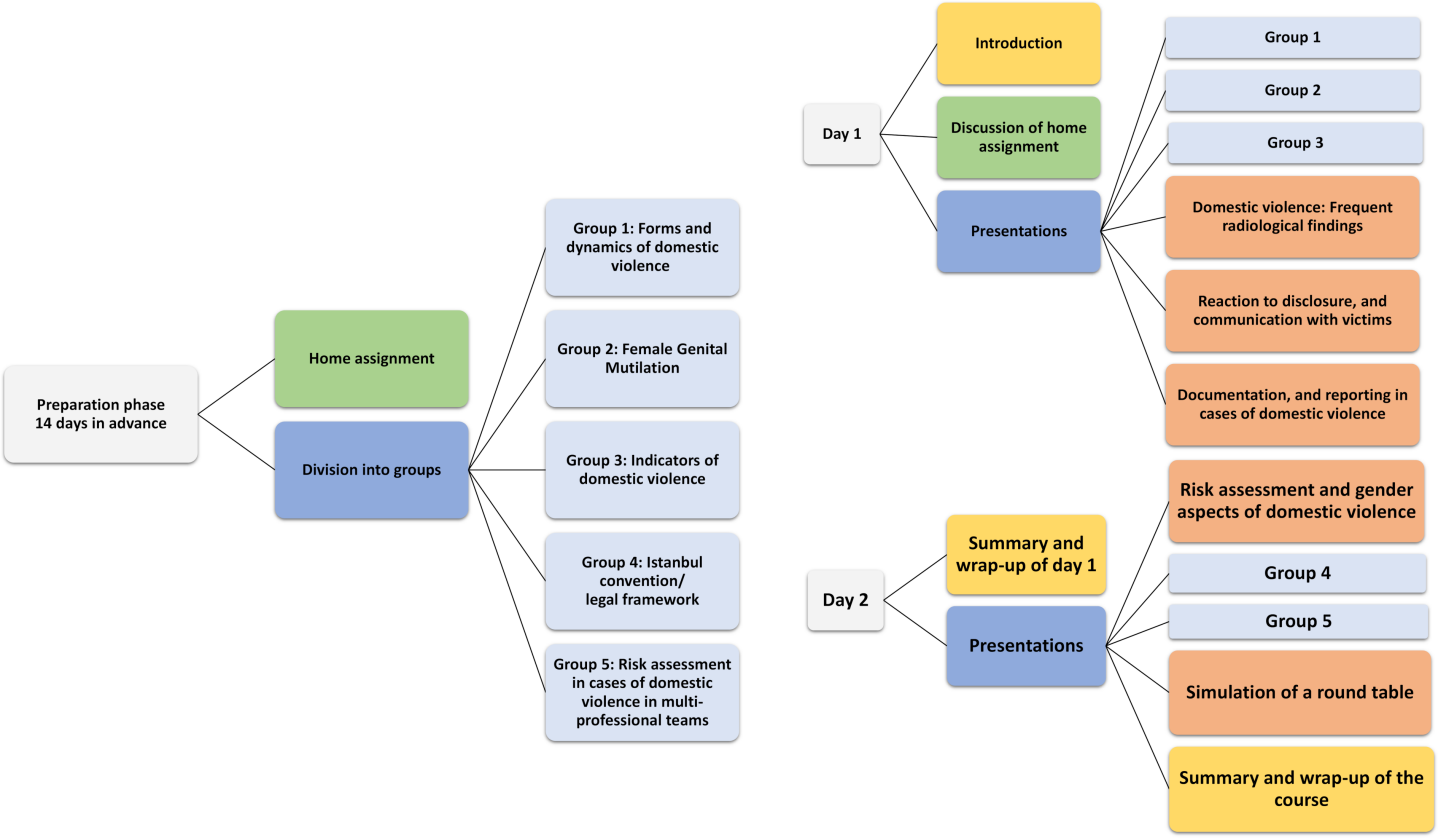
Structure of the compulsory elective course on DV based on the IMPRODOVA training platform

Prior to the elective course, the students had to complete a home assignment and had to work in groups on specific modules (see figure 1 (Fig. 1)) creating presentations for the whole learning group. Including preparation time for the home assignment and group presentations the duration of the course corresponded to a total of 28 semester hours, where one semester hour equals 45 minutes. The course is being held since summer 2020 once a term and elicits high interest among the students. 

Students had to send in the home assignment with a maximum of two pages prior to the course to the tutor and had to answer the following questions regarding three quotes related to DV (see attachment 2 ):

1. What do these quotations ring in you? What do they say about domestic violence?
2. Think about the ways in which you may have encountered the term ‘domestic violence and abuse’ so far. How do you define domestic violence and abuse? What does the terms mean for you and are you aware of any other terms describing the same phenomenon?

In addition, five groups were formed based on the preferences of the students covering the following topics (see table 1 (Tab. 1)):

- Group 1: Forms and dynamics of DV
- Group 2: Female Genital Mutilation (FGM)
- Group 3: Indicators of DV
- Group 4: Istanbul convention/legal framework
- Group 5: Risk assessment in cases of DV in multi-professional teams

The input of the various groups needed to be sent in at least three days before the course in order to ensure correct presentation of the various contents, to avoid duplication and to give didactic advice for optimal online presentations.

Students were told that they should use material from the IMPRODOVA training platform for their input [https://training.improdova.eu/en/]. In addition to the theoretical framework, they were told – depending on the group’s topic – to develop case studies and a training video, design campaign posters, prepare and facilitate a panel discussion, or conduct an interview with a frontline responder. Some of this was added to the training platform later (e.g., an interview on FGM).

As it can never been ruled out that participants themselves have been victims or witnesses of DV, this was addressed in advance with students. A private conversation was held with three out of the 37 students completing the courses after they submitted their home assignments. The students were offered to drop out of specific parts if the content would be too traumatic for them. No one took up the offer.

A special focus was put on sex and gender aspects in DV risk assessment, as sex, gender, own mind-sets, and expectations may not only have an impact on how one speaks with women and with men, respectively (e.g., strong voice, holding eye contact), but they can also influence how one assesses the risk, the aspects recognised as being significant (e.g., who started the incident), and how one perceives the victim, as well as how one is perceived by the victim [10], [12]. Therefore, the awareness of sex and gender aspects, particularly gendered perceptions and biases in DV is of major importance to frontline responders [6], [10]. 

The elective course is based on an innovative teaching concept, in which the focus is not only on teaching facts, but on the critical assessment of complex situations in everyday medical practice [13]. This transformative participatory approach ensured that students got more engaged with the subjects in a sustainable way by creating free spaces for reflective, experimental and critical teaching. Group discussion should encourage self-reflection and allows stereotypes and prejudices to be recognised. Students should exchange their expectations of their own future role as physicians, but also express their anxieties and doubts. The course was conducted virtually as an interactive seminar with a maximum number of 16 students to enable good learning atmosphere and to foster mutual discussion.

#### 2.3. Evaluation of the course “domestic violence in an international context”

##### 2.3.1. Knowledge assessment prior to the course

Knowledge assessments on DV before training were conducted on 54 medical students at the German Universities of Muenster (n=37) and Luebeck (n=17) (see attachment 3 ). Muenster medical students who had participated in the clinical compulsory elective course “domestic violence in an international context”, on October 24-25, 2020, and on June 19-20, 2021, were surveyed. The surveys were conducted prior to the elective course in the periods from October 14-23, 2020, and from April 1 and June 15, 2021, respectively. In Luebeck, medical students who had registered for a 2h workshop “medicine, don’t look away! – domestic violence in an international context from different perspectives”, on December 3, 2020, participated in the survey as well. The survey was conducted prior to the event between November 23, 2020, and December 3, 2020.

On average, the students were in their 4th year of medical school. The average age of the students was 25 years (M=24.98; SD=3.89). 46 of the 54 (85%) students were female. 20 of the 54 (37%) students stated that they had some form of professional experience with DV. Only four of the 54 (7%) students reported that they had received curricular training on DV of a few hours. Two of the 54 (4%) students had previously participated in any training on DV of a few hours and one student (2%) in a training on DV of a few weeks.

##### 2.3.2. Post-evaluation of the course

28 of the 37 students in Muenster who had participated in the pre-surveys took part in the post-surveys (see attachment 4 ). The evaluation took place after the end of the course in the periods from October 25, 2020, to November 8, 2020, and from June 20, 2021, to July 4, 2021, respectively. The purpose of these surveys was to evaluate and further optimise the elective course, as well as to assess its contribution to students’ learning and competence progress in DV.

The surveys were created using Questback’s online survey research tool EFS (Enterprise Feedback Suite) Survey (Fall 2019) [https://www.questback.com/]. The tool made it possible to create controlled implementation conditions. This was primarily achieved through the standardised questionnaire. In terms of content, the following topics were selected for the student surveys:

- Socio-demographic data and study biography
- Assessment of own attitudes towards DV
- Assessment of own knowledge about DV
- Assessment of own interests in DV
- Assessment of own competence regarding DV

Methodologically, the surveys consisted of single-choice questions, multiple-choice questions and assessment questions (see attachment 3 ). While questions on socio-demographic data and the course of the study were formulated as questions, assessment questions were formulated as statement sentences. Participants who had taken part in the knowledge assessments prior to the course could skip the socio-demographic questions in the post-surveys. In addition, students were not asked to list all terms they associate with the different forms of DV again after completing the course (see attachment 5 ). Participation in the surveys was voluntary and anonymous. Individuals who participated in the survey are not identifiable, the information was analysed in aggregated, not individual form.

##### 2.3.3. Analysis

Within the framework of the survey, a multi-level Likert scale was used for each question. The data of the individual items of a Likert scale are ordinal or rank-scaled. As the individual distances within the expressions cannot be considered equal per se, generally, results can only be presented using the position parameters mean and median for each item. Since in the present study it is guaranteed that the Likert scale is formulated symmetrically and the scale points could thus be interpreted as equally spaced by all respondents, a calculation of the averaged mean value was permissible and it was possible to add up the scores of the individual item responses of the Likert scales, resulting in a sum score for the overall scale. This value is clearly to be regarded as metric (interval-scaled). Consequently, mean values as well as standard deviation were calculated from the sum values, on which the further statistical evaluation procedures were based [14], [15]. Those were calculated separately for the samples each (Muenster and Luebeck) and are reported for the different time points (pre and post). The results described below were calculated only for the Muenster samples, since no data on the time point after the seminar were available for the Luebeck students as participation in the evaluation was voluntary and no one from Luebeck took part in the post-survey.

The means were calculated for the descriptive presentation and are also reported. To assess the statistical significance of the mean differences before and after the seminar for the Muenster students, paired sample t-tests were calculated for those where information was available at both time points. In addition, effect sizes were calculated using Cohen’s d. The mean difference was calculated in relation to the pooled standard deviation. That allowed all data to be used, regardless of whether information was available at both times or only at one time.

## 3. Results

### 3.1. Knowledge assessment on DV

In the knowledge assessments, the students were asked to list all the terms they associate with the different forms of DV (see attachment 5 with corresponding frequencies of terms being listed). After categorising the terms, it became apparent that DV was mostly associated (91.7%) with physical violence by the students before a corresponding training. Other forms of DV were mentioned less frequently (<50%). In addition, potential causes (e.g., power imbalances between intimate partners) and consequences (e.g., physical injuries, shame) of DV were mentioned. Students considered the intervention of DV cases as an important part of the work of physicians and are motivated to work with victims of DV, but it is difficult for them to ask patients about DV (see table 2 (Tab. 2)). This is not surprising, as most students (see 2.3.1) have not received any curricular education or training on DV at both universities. Overall, students have a strong interest in the various issues related to DV, but the majority of students do not feel (well) informed about them (see table 3 (Tab. 3)). Accordingly, the majority of students also do not consider themselves competent regarding the above-mentioned topics. Finally, against the background of these results, it becomes clear that there is a great need for training on DV among medical students and that there is also interest on the part of the students.

#### 3.2. Post-survey results after completing the training 

After comparing the post-course responses with those prior to the course it could be shown that the elective course using the IMPRODOVA training platform with its teaching materials, has contributed to a significant attitude change, learning and competence progress of the students in all subject areas (see table 2 (Tab. 2), table 3 (Tab. 3) and table 4 (Tab. 4)). The students liked the training materials offered on the training platform, especially the case studies.

##### 3.2.1. Comparison of students' attitudes before and after the elective course

Basic attitudes and interests of the students (“To what extent do you agree or disagree with the following statements?” and “How interested are you in the following topics?” did mostly not change after completion of the study course. There were however two noteworthy exceptions: “Intervention in cases of domestic violence is an important part of my work.” (“To what extent do you agree or disagree with the following statements?”) and “It is difficult for me to ask patients about domestic violence.” (“To what extent do you agree or disagree with the following statements?”). Students perceived intervention in cases of DV as a more important part of their future work than before (*d*=0.88; *t*(26)=3.12, *p*=.004) and they found it easier to ask about DV after completion of the student course (*d*=1.03; *t*(26)=2.55, *p*=.017) (see table 2 (Tab. 2)).

##### 3.2.2. Level of information

Prior to the course, the majority of students indicated that they were not or hardly informed about tools identifying DV victims and assessing risks related to DV. After the course, all students indicated to be well or even very well informed and felt significantly more informed than before (*d*=2.80–3.90, *t*(26)=9.48–12.30, *p*<.001). Similarly, students felt better informed about interagency cooperation of first responders to DV after the course (*d*=2.80, *t*(27)=9.48, *p*<.001) (see table 3 (Tab. 3)).

##### 3.2.3. Competences of the students

**Figure 2:**
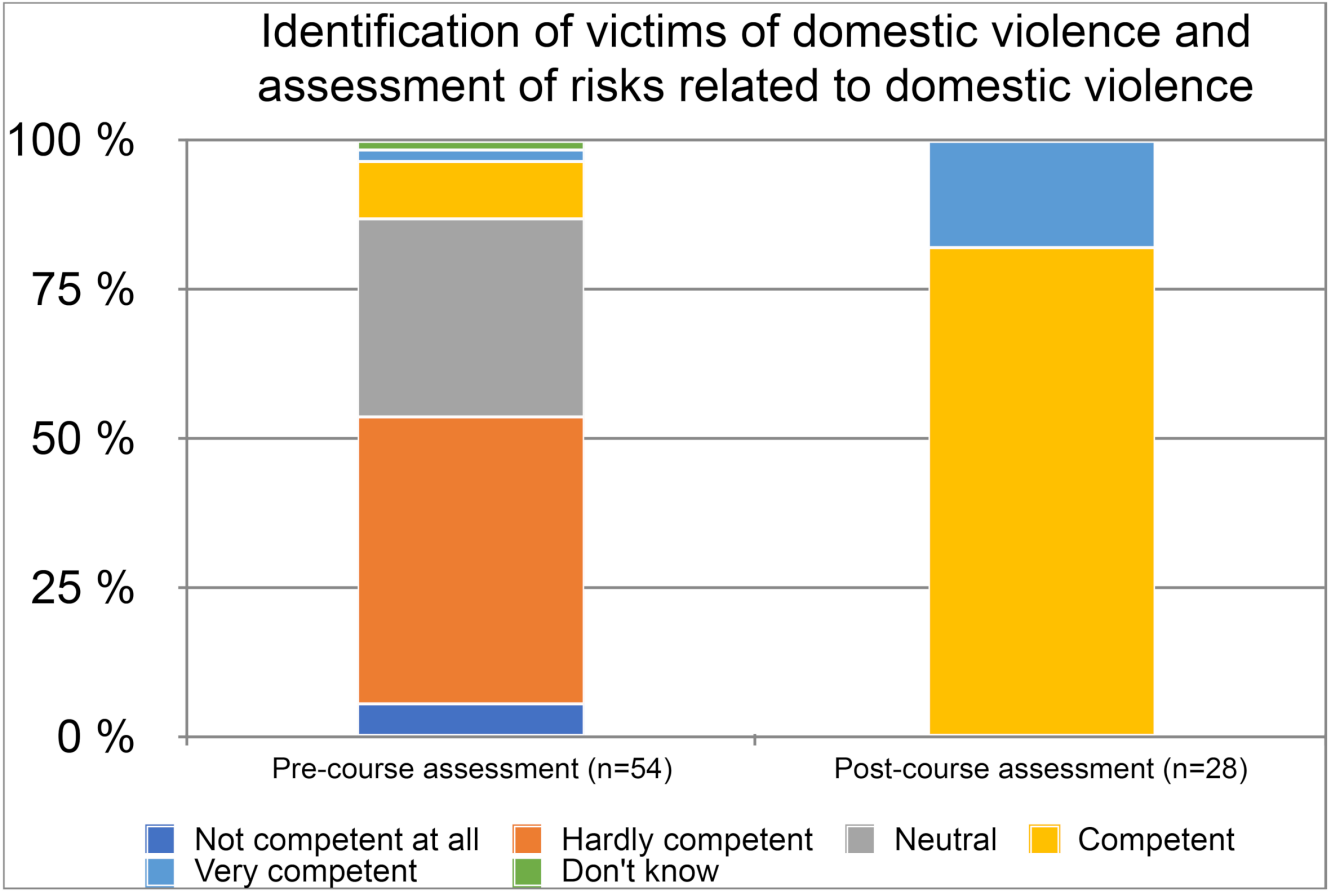
Level of competence regarding regional cooperation with other first responders in cases of DV

Data presented in table 4 (Tab. 4) (see also figure 2 (Fig. 2)) indicate that students felt much more competent in those subject areas listed after the elective course (*d*=1.90–2.65), *t*(26)=7.57–13.24, *p*<.001). Two areas need to be highlighted as we consider them as very important for medical DV frontline responders: before the course more than half of the students considered themselves hardly competent in identifying victims of DV and assessing risks related to DV (M_Luebeck_=2.47, M_Muenster_=2.56) or working with other frontline responders on cases (M_Luebeck_=2.65, M_Muenster_=2.25). After the course, all students felt competent or very competent regarding identification (*d*=2.64 , *t*(27)=7.78, *p*<.001) and cooperation (*d*=1.54, *t*(27)=7.57, *p*<.001).

## 4. Discussion

In this project report, we present a newly developed clinical compulsory elective course on DV and its evaluation. To our knowledge, the Medical School at the University of Muenster offers the first teaching program on DV for medical students in Germany. Internationally, there is also only a limited number of courses offered on this topic for physicians (e.g., in Australia [16]). Knowledge assessment about DV revealed that there is a great need for training on DV among medical students. Students believe that knowledge on DV in medicine is important, but at the same time feel that their level of knowledge and competence in DV is low.

The theoretical framework and case studies of the elective course were rated by most students as being informative and useful. The structure as well as the contents of the course seem to be well suited to teach knowledge on DV adequately. The students evaluated the elective course as being relevant for their future medical practice.

Nevertheless, we only collected the students’ personal assessment of their own level of knowledge and there is no objective data on knowledge gain in terms of a test or similar. Likewise, it would have been desirable to collect data on the extent to which the knowledge is still available at a further measurement point – for example a few weeks after the course. An assessment of the implementation of the acquired knowledge in practice from the perspective of the students or the supervisors would also allow further statements about the success of the seminar.

The statistical analyses of the evaluation assumed that the distances between the points on the Likert scale are equal, which – as mentioned before – would allow the treatment of the ordinal data as metric. We assumed this in the context of the study due to the numerical anchoring, nevertheless it would make the evaluation more stable if a previous survey on an independent sample had been possible about this.

As participation in this course was voluntarily and not part of the mandatory medical curriculum, we were only able to raise awareness among a small group of students about this important topic. Other limitations of our evaluation are that students may have registered for the course who were already interested in or sensitised to this topic. As the evaluation was voluntary students, who were interested in this topic and particularly liked the course may have been more likely to participate in this evaluation. In addition, we have no post-data from the Luebeck students, as no one was willing to participate in the post-evaluation.

Due to the unequal gender ratio within the courses, statements are limited as to what extent the results apply equally to men and women. Since the gender ratio in the course does not reflect that of the study programme, the question arises as to whether the statements are limited to mainly female participants and why the interest of male students in the topic is rather low. Certain gender aspects could thus only be addressed to a limited extent in the context of the courses, which may have minimised the learning gain.

Based on our evaluation, it is of major importance to raise awareness among all medical students. The huge interest of students in Muenster to participate in this elective course reflects that they appreciate the opportunity to openly discuss and express their own thoughts about DV. At the same time, it shows how little students know about this topic and how big the need for information about domestic violence in medicine is.

## 5. Conclusions

Our evaluation results confirmed that existing knowledge on DV in medicine is not sufficient and teaching materials are lacking. Our clinical compulsory elective course for medical students seems to be well suited to close this gap and teach knowledge on DV and we recommend to integrate aspects of how to deal with DV in medicine as part of a mandatory curriculum.

## Funding

The IMPRODOVA project has received funding from the European Union’s Horizon 2020 research and innovation programme under grant agreement No 787054. We would like to thank our IMPRODOVA partners, without whose input the training platform would not have been possible.

## Competing interests

The authors declare that they have no competing interests. 

## Supplementary Material

Exemplary course agenda which can be modified or amended at any time since it is a living document

Home assignment

This questionnaire was used to assess the knowledge of students from Muenster and Luebeck on DV prior to our training

This questionnaire was used to assess the knowledge of students from Muenster about DV after completion of the elective course

Lists of terms and their frequency being mentioned students associated with the different forms of DV in the knowledge assessments

## Figures and Tables

**Table 1:**
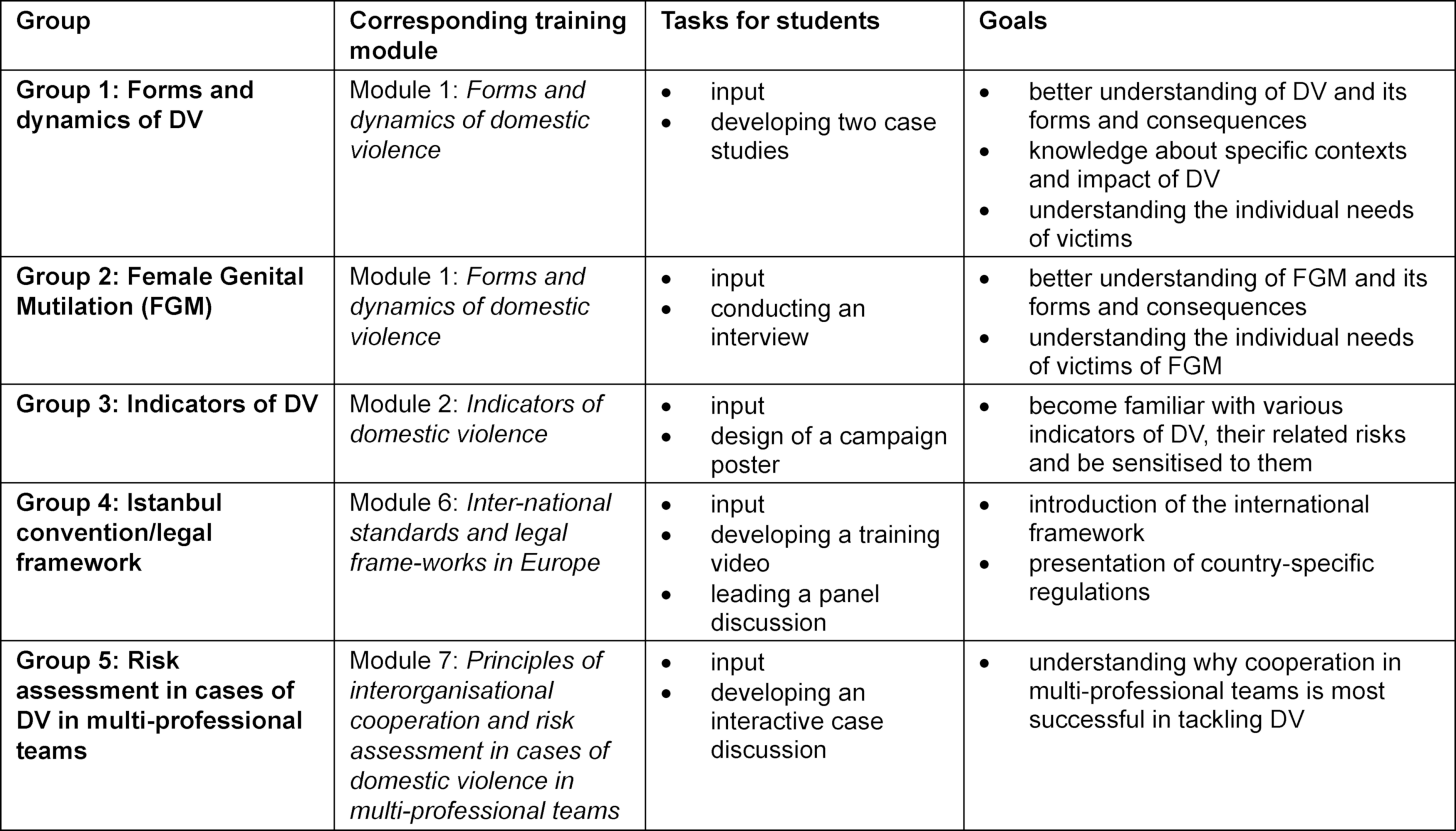
In the elective course on DV were medical students assigned to the following groups

**Table 2:**
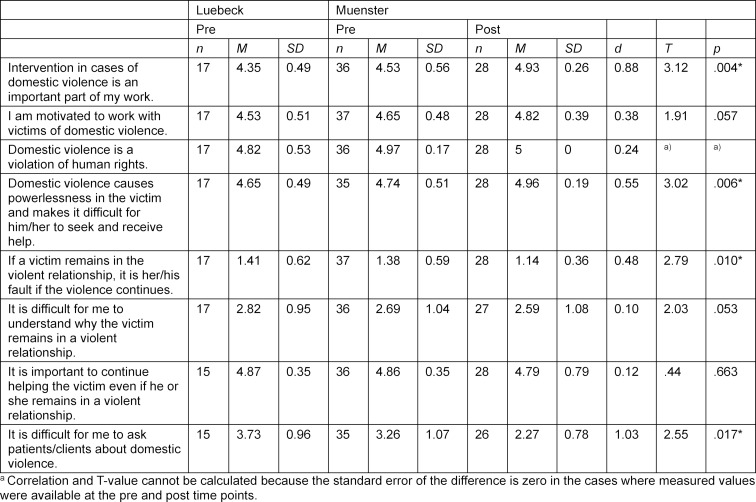
Attitudes from students in Muenster and Luebeck before and in Muenster after the elective course measured on a 5-Point-Likert-Scale (1=very high disagreement up to 5=very high agreement) regarding certain topics. Since a Likert-scale is classified as metric, the data allows calculations of the mean and standard deviation per item. Means, standard deviations, Cohen’s d and results from paired t-tests are presented. The number of participants fluctuates per Item because the rating “don´t know” was not taken into consideration. Paired t-Tests were only calculated in the cases where measured values were available at the pre and post time points (n=27). *p<.05 = significant change of scores pre and after the course.

**Table 3:**
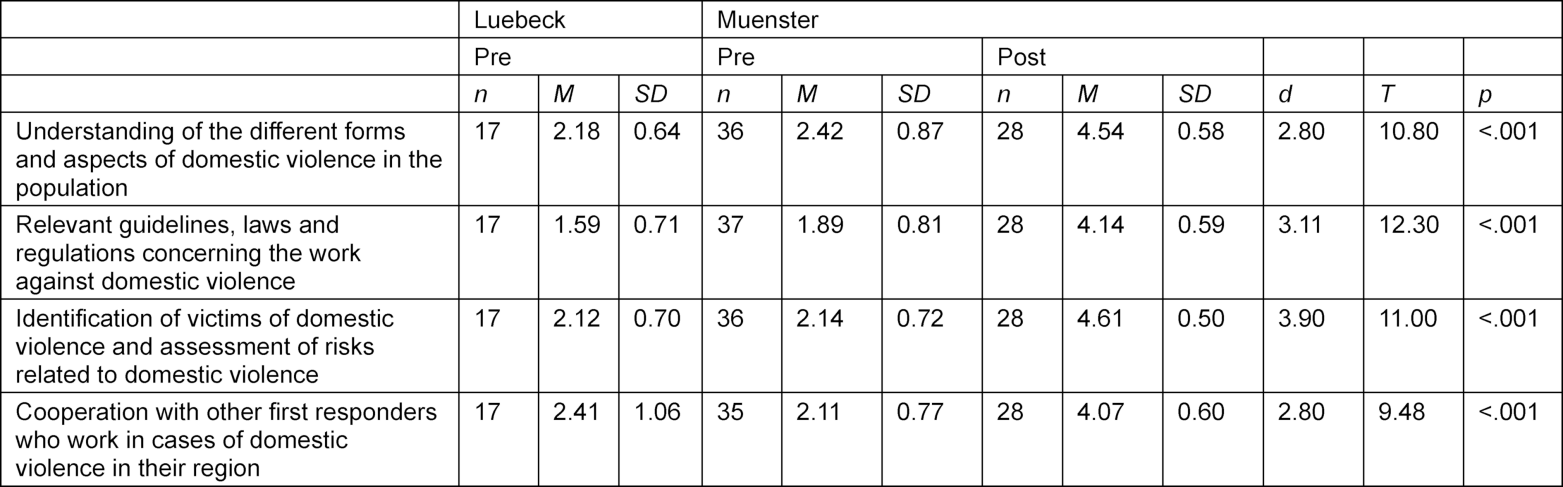
Level of feeling informed from students in Muenster and Luebeck before and in Muenster after the elective course measured on a 5-Point-Likert-Scale (1=not at all up to 5=very well) regarding certain topics. Since a Likert-scale is classified as metric, the data allows calculations of the mean and standard deviation per item. Means, standard deviations, Cohen’s d and results from paired t-tests are presented. The number of participants fluctuates because the ratings “don´t know” was not taken into consideration. Paired t-Tests were only calculated in the cases where measured values were available at the pre and post time points (n=27).

**Table 4:**
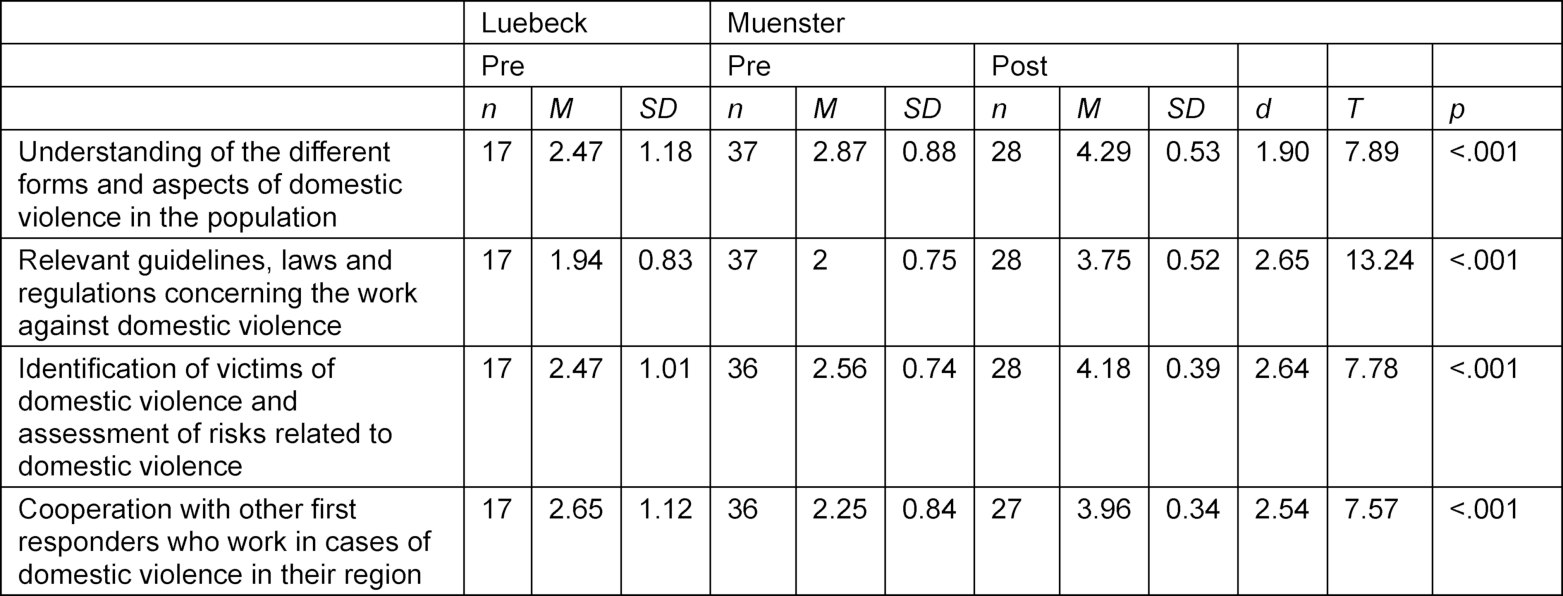
Level of competence from students in Muenster and Luebeck before and in Muenster after the elective course measured on a 5-Point-Likert-Scale (1=not at all up to 5=very well) regarding certain topics. Since a Likert-scale is classified as metric, the data allows calculations of the mean and standard deviation per item. Means, standard deviations, Cohen’s d and results from paired t-tests are presented. The number of participants fluctuates because the rating “don´t know” was not taken into consideration. Paired t-Tests were only calculated in the cases where measured values were available at the pre and post time points (n=27).
