# Supplementary material for: Introduction and evaluation of a clinical compulsory elective course on domestic violence
Source: GMS J Med Educ. 2022 Nov 15;39(5):Doc56. doi: 10.3205/zma001577 (PMC9733485; doi:10.3205/zma001577)
Supplement: Exemplary course agenda which can be modified or amended at any time since it is a living document [file JME-39-56-s-001.pdf]

Attachment 1: Exemplary course agenda which can be modified or amended at any time since it is a living document.

Day 1 of the student course:

- **10.00 am – 10.15 am** Introduction of participants, introduction into the topic
- **10.15 am – 10.45 am** Discussion of the home assignment
- **10.45 am – 11.45 am** Group 1: *Forms and dynamics of DV*
  Input by students and developing of two case studies using material of Module 1 of the IMPRODOVA training platform [https://training.improdova.eu/en/training-modules-for-the-health-sector/module-1-forms-and-dynamics-of-domestic-violence/]
- **11.45 am – 12.00 am** *coffee break*
- **12.00 am – 0.45 am** Group 2: *Female Genital Mutilation*
  Input by students and conduction of an interview with a specialist from the German Female Genital Mutilation Education Portal KUTAIRI [https://www.kutairi.de/] using material of Module 1 of the IMPRODOVA training platform [https://training.improdova.eu/en/training-modules-for-the-health-sector/module-1-forms-and-dynamics-of-domestic-violence/]
- **0.45 am – 1.30 pm** *lunch*
- **1.30 pm – 2.15 pm** Group 3: *Indicators of DV*
  Input by students and design of a campaign poster using material of Module 2 of the IMPRODOVA training platform [https://training.improdova.eu/en/training-modules-for-the-health-sector/module-2-indicators-for-domestic-violence/]
- **2.15 pm – 3 pm** *Domestic violence: Frequent radiological findings*
  Input by a radiologist using material of Module 2 of the IMPRODOVA training platform [https://training.improdova.eu/en/training-modules-for-the-health-sector/module-2-indicators-for-domestic-violence/#Domestic-violence-Frequent-radiological-findings]
- **3 pm – 3.15 pm** *break*
- **3.15 pm – 4.30 pm** *Reaction to disclosure, and communication with victims*
  Input by the course leader and interactive case discussion using material of Module 3 of the IMPRODOVA training platform [https://training.improdova.eu/en/training-modules-for-the-health-sector/module-3-communication-in-cases-of-domestic-violence/]
- **4.30 pm – 4.45 pm** *Documentation, and reporting in cases of DV*

Day 2 of the student course:

- **10.00 am - 10.30 am** *Knowledge assessment and wrap-up by using a quiz*
- **10.30 am – 11.40 am** *Risk assessment and gender aspects of risk assessment*
- Input by the course leader and interactive case discussion using material of Module 5 of the IMPRODOVA training platform [https://training.improdova.eu/en/training-modules-for-the-health-sector/module-5-risk-assessment-and-safety-planning/]
- **11.40 am – 11.50 am** *coffee break*
- **11.50 am – 12.50 am** Group 4*: Istanbul convention, legal framework*
  Input by students and developing of a training video, and panel discussion using material of Module 6 of the IMPRODOVA training platform [https://training.improdova.eu/en/training-modules-for-the-health-sector/module-6-international-standards-and-legal-frameworks-in-europe/]
- **12.50 am – 1.45 pm** *lunch*
- **1.45 pm – 2.30 pm** Group 5: *Risk assessment in cases of DV in multi-professional teams*
  Input by the course leader and interactive case discussion using material of Module 7 of the IMPRODOVA training platform [https://training.improdova.eu/en/training-modules-for-the-health-sector/module-7-principles-of-interorganisational-cooperation-and-risk-assessment-in-cases-of-domestic-violence-in-multi-professional-teams/]
- **2.30 pm – 2.45 pm** *"Round table" Female Genital Mutilation*
  Input by students using material of the German Female Genital Mutilation Education Portal KUTAIRI [https://www.kutairi.de/]
- **2.45 pm – 3. pm** *coffee break*
- **3 pm – 4.30 pm** *Simulation of a "round table"- cooperation of multi-professional teams by discussing a case of domestic violence*
  Input by the course leader and interactive case discussion using material of Module 7 of the IMPRODOVA training platform [https://training.improdova.eu/en/training-modules-for-the-health-sector/module-7-principles-of-interorganisational-cooperation-and-risk-assessment-in-cases-of-domestic-violence-in-multi-professional-teams/]
- **4.30 pm – 4.50 pm** *Wrap-up by using "Ficts and Facts"*
  [https://training.improdova.eu/en/training-materials-for-the-health-sector/ficts-and-facts/]
- **4.50 pm** Feedback round and farewell
