# Supplementary material for: Introduction and evaluation of a clinical compulsory elective course on domestic violence
Source: GMS J Med Educ. 2022 Nov 15;39(5):Doc56. doi: 10.3205/zma001577 (PMC9733485; doi:10.3205/zma001577)
Supplement: Home assignment [file JME-39-56-s-002.pdf]

Attachment 2: Home assignment

The following tasks serve as an introduction to the course on domestic violence. Please summarize your answers in 1-2 pages and send the home assignment the latest until (date) to (e-mail address of lecturer).

‘So many people don’t understand what domestic violence is about. It’s not all about being beaten up, it’s also not allowing you to see your friends; not allowing you your own money; it’s controlling your life in every possible way; no one can see it.‘

‘Helping a victim of domestic violence means laying down the base of a house. Only this will allow her to step out of the situation. It means to map the past and to map the present in terms of the roots of violence in the family and all the consequences of violence in the present.‘

‘Intimate partner violence is not the specificity of the poor and uneducated, like many people misbelieve. The wife of a well-known politician was sheltered here. Upper middle-class family. The husband was a well-respected community leader and a churchman. Some people knew what was happening behind closed doors. Kids had to witness how the father raped the mother. He even peed on the kids as a punishment for misbehaving. The man was so influential in his city that the Guardianship office refused to help when they realized that his name is involved. It was almost hopeless for the wife to escape from her prison, because most people would not believe her and those who believed her didn’t dare to help her.‘

Tasks

1) What do these quotations ring in you? What do they say about domestic violence?

2) Think about the ways in which you may have encountered the term ‘domestic violence and abuse’ so far. How do you define domestic violence and abuse? What does the terms mean for you and are you aware of any other terms describing the same phenomenon?
