# Supplementary material for: Introduction and evaluation of a clinical compulsory elective course on domestic violence
Source: GMS J Med Educ. 2022 Nov 15;39(5):Doc56. doi: 10.3205/zma001577 (PMC9733485; doi:10.3205/zma001577)
Supplement: This questionnaire was used to assess the knowledge of students from Muenster and Luebeck on DV prior to our training [file JME-39-56-s-003.pdf]

**Attachment 3:** This questionnaire was used to assess the knowledge of students from Muenster and Luebeck on DV **prior to our training**.

This survey is part of IMPRODOVA, a research and innovation project funded by the European Union's Horizon 2020 programme. Part of IMPRODOVA – “Improving Frontline Responses to High Impact Domestic Violence” (www.improdova.eu), is an optimised response to domestic violence by frontline responders in eight European countries.

The purpose of this survey is to evaluate IMPRODOVA training materials developed for social and health care professionals, medical students, physicians, and police officers. Your participation in the survey is therefore very important for the further optimisation of these IMPRODOVA training materials!

**You will soon be attending a clinical course on domestic violence. In advance we would like to collect some information.**

Participation in the survey is voluntary and anonymous. Persons who take part in the survey are not identifiable, the information is analysed in an aggregated, non-individualised form. Participants have the option of cancelling the survey at any time without explanation or consequences of any kind.

Please first create an individual code so that we can anonymously compare the questionnaires before and after the seminar.

1st digit: First letter of your mother's first name (e.g., Gerda - G)
2$^{nd}$ & 3$^{rd}$ digit: Your month of birth (e.g., August - 08)
4$^{th}$ digit: Last letter of your place of birth (e.g., Muenster - M)
Example code: G08M

1. In which semester are you currently studying medicine?

2. How old are you?

Attachment 3 to: Juszczyk P, Sondern L, Pfleiderer B. *Introduction and evaluation of a clinical compulsory elective course on Domestic Violance.* GMS J Med Educ. 2022;39(5):Doc56. DOI: 10.3205/zma001577

3. What is your gender?

Man

Woman

Non-binary

I prefer not to respond.

4. Have you had previous work experience with domestic violence?

Yes

No

5. Have you already received curricular training on domestic violence?

Yes

No

6. Have you participated in any training on domestic violence previously?

Yes, for a few hours (e.g., a lecture)

Yes, for a few days (e.g., a conference)

Yes, for a few weeks (e.g., a course)

No

7. Please list here all terms you associate with the different forms of domestic violence.

8. To what extent do you agree or disagree with the following statements?

| | 1 = Strongly disagree | 2 = Disagree | 3 = Neutral | 4 = Agree | 5 = Strongly agree | 0 = Don't know |
|---|---|---|---|---|---|---|
| Intervention in cases of domestic violence is an important part of my work. | | | | | | |
| I am motivated to work with victims of domestic violence. | | | | | | |
| Domestic violence is a violation of human rights. | | | | | | |
| Domestic violence causes powerlessness in the victim and makes it difficult for him/her to seek and receive help. | | | | | | |
| If a victim remains in the violent relationship, it is her/his fault if the violence continues. | | | | | | |
| It is difficult for me to understand why the victim remains in a violent relationship. | | | | | | |
| It is important to continue helping the victim even if he or she remains in a violent relationship. | | | | | | |
| It is difficult for me to ask patients/clients about domestic violence. | | | | | | |

Attachment 3 to: Juszczyk P, Sondern L, Pfleiderer B. *Introduction and evaluation of a clinical compulsory elective course on Domestic Violance.* GMS J Med Educ. 2022;39(5):Doc56. DOI: 10.3205/zma001577

9. How well informed do you feel about the following topics in general?

| | 1 = Not informed at all | 2 = Hardly informed | 3 = Neutral | 4 = Well informed | 5 = Very well informed | 0 = Don't know |
|---|---|---|---|---|---|---|
| The different forms and aspects of domestic violence in the population (including special groups such as various minorities and older people) | | | | | | |
| Relevant guidelines, laws and regulations concerning the work against domestic violence | | | | | | |
| Tools to identify domestic violence and assessment of risks related to domestic violence | | | | | | |
| Other frontline responders who work in cases of domestic violence in their region (e.g., authorities, agencies) | | | | | | |

10. How interested are you in the following topics?

| | 1 = Not interested at all | 2 = Hardly interested | 3 = Neutral | 4 = Interested | 5 = Very interested | 0 = Don't know |
|---|---|---|---|---|---|---|
| The different forms and aspects of domestic violence in the population (including special groups such as various minorities and older people) | | | | | | |
| Relevant guidelines, laws and regulations concerning the work against domestic violence | | | | | | |
| Tools to identify domestic violence and assessment of risks related to domestic violence | | | | | | |
| Other frontline responders who work in cases of domestic violence in their region (e.g., authorities, agencies) | | | | | | |

11. How competent do you consider yourself to be in the following areas?

| | 1 = Not competent at all | 2 = Hardly competent | 3 = Neutral | 4 = Competent | 5 = Very competent | 0 = Don't know |
|---|---|---|---|---|---|---|
| Understanding of the different forms and aspects of domestic violence in the population (including specific groups such as various minorities and older people) | | | | | | |
| Relevant guidelines, laws and regulations concerning the work against domestic violence | | | | | | |
| Identification of victims of domestic violence and assessment of risks related to domestic violence | | | | | | |
| Cooperation with other frontline responders who work in cases of domestic violence in their region | | | | | | |

Thank you very much for your participation in this evaluation. After the elective course, you will receive another link asking you to fill out this evaluation as well.

You can now close the window.
