# Supplementary material for: Introduction and evaluation of a clinical compulsory elective course on domestic violence
Source: GMS J Med Educ. 2022 Nov 15;39(5):Doc56. doi: 10.3205/zma001577 (PMC9733485; doi:10.3205/zma001577)
Supplement: Lists of terms and their frequency being mentioned students associated with the different forms of DV in the knowledge assessments [file JME-39-56-s-005.pdf]

Attachment 5: Lists of terms and their frequency being mentioned students associated with the different forms of DV in the knowledge assessments (see attachment 2).

**Category**	**Terms**
Physical violence (34)	Hitting (9); Abuse (7); Murder/Femicide (3); Biting (1); Burning (1); Holding (1); Physical assaults (1)
Psychological/Emotional/Mental violence (17)	Suppression (7); Control (7); Threatening (7); Insulting (8); Psycho terror (4); Social isolation (4); Verbal violence (6); Stalking (4); Observing (1); Humiliation (7); Locking (2); Intimidation (3); Coercion (1); Emotional dependency (1); Guilty feelings (1); Ignorance (1); Destruction of objects (1); Manipulation (2); Jealousy (1); Aggression (1); No privacy (1); Words (1); Shouting (1); Lying (1); Mobbing (1); Gaslighting (1); Blackmail (2); Harassment (1)
Sexual/Sexualised violence (14)	Sexual abuse (9); Rape (8); Sexual harassment (1)
(Socio-)Economic/Financial violence (4)	Financial withdrawal (1); Economic dependency (1); Work ban (1); Withholding of wages (1); Financial control (2)
Neglect (4)	
Violence against women and children (5)	Infantile shaking trauma (1); Childhood trauma (1); Child traumatisation (1)
Multidisciplinary cooperation	Women's shelter (4); White ring (1); Psychologists (1); Psychiatrists (1); Forensic ambulance (1); Forensics (2); Contact points (1); Cooperation with the police (1); Emergency hotlines (1); Intervention (1); Youth welfare (1); Evidence (1)
Consequences of DV	Shame (7); Isolation (5); Anxiety (6); Dependency (2); Powerlessness (2); Silence (1); Excessive demand (1); Psychological consequences (1); Pain (2); Insecurity (1);

Attachment 5 to: Juszczyk P, Sondern L, Pfleiderer B. *Introduction and evaluation of a clinical compulsory elective course on Domestic Violance.* GMS J Med Educ. 2022;39(5):Doc56. DOI: 10.3205/zma001577

	Despair (1); Love (1); Being trapped (1); Long-term consequences (1); Helplessness (1); Fainting (1); Hopelessness (1); Self-doubts (1); Mourning (1); Anger (4); Injuries (1)
Dynamics of DV	Power imbalance (7); Systematic (1); Endure (1); Protected by society (1); Anonymous (1); Hidden (1); Abuse of trust (1); Perpetrator-victim (1); High numbers of unknown cases (1); Hierarchy (1); Inequality (1); Dependency (4); Secret (3); Apologies (1); Professing to mend one's ways (1); Putting under pressure (2); Crisis (1); Suppression (6); Isolation (1); Withholding basic human rights (1); Deprivation of freedom of movement (1)
Constellations of DV	Victim (2), Perpetrator (1); Parents (2); Children (1); Women (1); Family (1); Relationship (2); Perpetrator-Victim (1); Mostly men as perpetrators and mostly women as victims (but not always!) (1)
Causes of DV	Loss of control (1); Alcohol (1); Aggression (1); Narcissism (1); “Honour” (1); COVID-19 (1)
